# Supplementary material for: Identification of photoperiod-induced specific miRNAs in the adrenal glands of Sunite sheep (Ovis aries)
Source: Front Vet Sci. 2022 Jul 22;9:888207. doi: 10.3389/fvets.2022.888207 (PMC9354845; doi:10.3389/fvets.2022.888207)
Supplement: Supplementary file 1 [file Data_Sheet_1.ZIP › Supplementary file/miRDeep2 file.zip!/miRDeep2 file/Know_Structure/LP42A2/oar-mir-485.pdf]

miRBase precursor : oar-mir-485
Total read count : 1675
oar-miR-485-5p read count : 722
oar-miR-485-3p read count : 952
remaining reads : 1

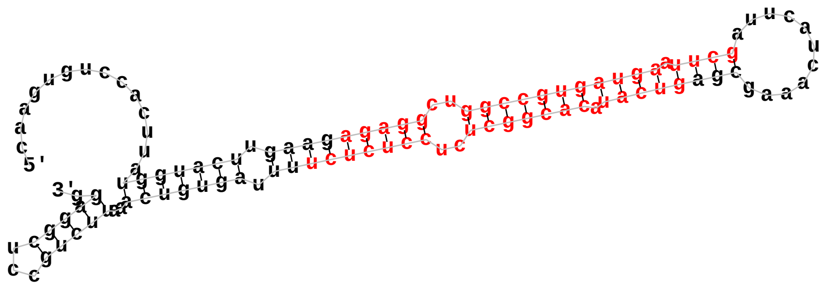

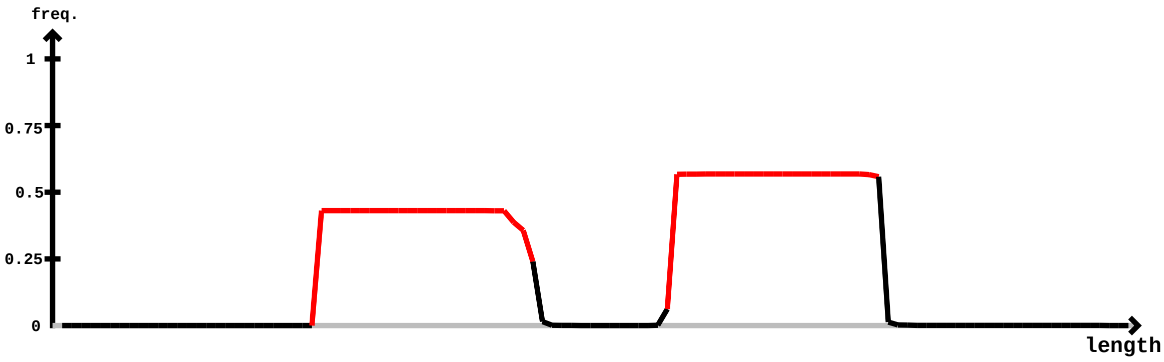

oar-miR-485-5p

oar-miR-485-3p

```
5'- caaguguccacuuaugguacuugaagagaggcuggccgugaugaauucgauucaucaaagcgagucauacacggcucuccucucuuuuagugucaaauucugccucggagg   -3'   exp
    ((((((.(((....)))))))))((((((((..((((((((((.((((...........)))))))).)))))))..))))))))............(((((...)))))).   reads   mm   sample
    ..........................agaggcuggccgugauga...................................................................   1       0    seq
    ..........................agaggcuggccgugaugaau.................................................................   72      0    seq
    ..........................agaggcuggccgugaugaauu................................................................   51      0    seq
    ..........................agaggcuggccgugaugaauuc...............................................................   196     0    seq
    ..........................agaggcuggccgugaugaauucg..............................................................   377     0    seq
    ..........................agaggcuggccgugaugaauucga.............................................................   23      0    seq
    ..........................agaggcuggccgugaugaauucgau............................................................   1       0    seq
    ..........................agaggcuggccgugaugaauucgauuc..........................................................   1       0    seq
    .............................................................gagucauacacggcucuccucu............................   1       0    seq
    .............................................................gagucauacacggcucuccucucu..........................   1       0    seq
    ..............................................................agucauacacggcucuccucuc...........................   1       0    seq
    ..............................................................agucauacacggcucuccucucu..........................   99      0    seq
    ..............................................................agucauacacggcucuccucucuu.........................   2       0    seq
    ...............................................................gucauacacggcucuccucu............................   3       0    seq
    ...............................................................gucauacacggcucuccucuc...........................   12      0    seq
    ...............................................................gucauacacggcucuccucucu..........................   813     0    seq
    ...............................................................gucauacacggcucuccucucuu.........................   16      0    seq
    ...............................................................gucauacacggcucuccucucuuu........................   1       0    seq
    ...............................................................gucauacacggcucuccucucuuuu.......................   1       0    seq
    ................................................................ucauacacggcucuccucucu..........................   1       0    seq
    ..................................................................auacacggcucuccucucuuuu.......................   1       0    seq
    .....................................................................................uuuagugucaaauucugccucgg...   1       0    seq
```
